# Experimental Analysis of the Influence of the Application of TiN, TiAlN, CrN and DLC1 Coatings on the Friction Losses in an Aviation Internal Combustion Engine Intended for the Propulsion of Ultralight Aircraft

**DOI:** 10.3390/ma14226839

**Published:** 2021-11-12

**Authors:** Piotr Wróblewski, Robert Rogólski

**Affiliations:** 1 Faculty of Engineering, University of Technology and Economics H. Chodkowska in Warsaw, Jutrzenki 135, 02-231 Warsaw, Poland; 2 Faculty of Mechatronics, Armament and Aerospace, Military University of Technology, ul. Gen. Sylwestra Kaliskiego 2, 00-908 Warsaw, Poland; robert.rogolski@wat.edu.pl

**Keywords:** piston rings, friction losses, oil film, aircraft engine, coatings, ultralight aircraft

## Abstract

Currently, there are many methods of reducing the friction losses of the main components of an internal combustion piston engine. The operating conditions of internal combustion piston engines intended for the propulsion of ultralight aircraft differ significantly from those prevailing in the case of using these engines for the propulsion of vehicles. There are many studies on the influence of selected anti-wear coatings on the friction coefficients when using various lubricants, measured via tribometers. Unfortunately, the conditions obtained in the laboratory significantly differ from those prevailing in an engine operating under external conditions. The study investigated the influence of a change in the tribological parameters of TiN, TiAlN, CrN and DLC1 anti-wear coatings on the moment of resistance to the piston movement of an aircraft engine. The operating parameters of a real engine working in an aircraft were simulated. The main focus was on the coating layers of the sliding surfaces of the piston rings and the cylinder running surface. The properties of the coatings affect the correlation of the scale of the adhesion and cohesion phenomena of the oil to the opposite planes, and this determines the nature of the changes in the moment of resistance to engine motion. As it is commonly known, with an increase in the value of the maximum pressure of the working medium in the combustion chamber, the share of mixed friction in liquid friction increases, similar to the high oil temperatures occurring in aircraft engines. Therefore, there is a justified need to supplement the research in the field of analyzing the characteristics of the torque of resistance to motion for these engines, in particular in the field of the usable rotational speeds of the crankshaft. Applicable anti-wear systems based on selected coatings can significantly improve operational safety and noticeably reduce fuel consumption.

## 1. Introduction

Thermodynamic changes take place in the cylinder of aviation internal combustion piston engines, which translate into effective work. This work is transferred to the propeller drive with the given general efficiency. The piston together with the piston rings move in the cylinder, and adequate tightness must be maintained in order to neutralize the loss of charge as much as possible. The mechanical load on the pistons and piston rings in aviation internal combustion piston engines is much greater than in the case of engines intended for motor vehicle propulsion, where the maximum circulation pressure does not exceed 7 MPa. In aircraft engines, there is a much greater thermal load on the combustion chamber, which is related to the heat stream flowing through the walls surrounding the combustion chamber. A local assessment of the thermal load can be obtained by analyzing the temperature fields of the engine parts surrounding the working space of the combustion chamber. Knowledge of the temperature field makes it possible to determine the maximum temperature value and the temperature gradients affecting thermal stresses and deformations [1]. Considering the fact that aviation internal combustion engines operate at very different ambient temperatures and are mostly air-cooled (we are talking about small aircraft engines designed to drive drones and ultralight aircraft), their thermal load is stochastic. Sudden changes in the external temperature of the engine contribute to unforeseen local changes in the temperature of the engine oil, leading to a change in its dynamic viscosity. Therefore, these engines require the use of special materials, to maintain the required sealing of the combustion chamber; to maintain the designed stereometry of the working surfaces of the piston rings; piston and cylinder smoothness; and anti-wear coatings, reducing the frictional resistance under conditions of liquid and mixed friction. In addition, these materials are required to have high thermal conductivity, low thermal expansion, and low wear of working surfaces in the conditions of boundary and mixed friction [2]. An evaluation of these tribological parameters and the influence of selected anti-wear coatings on their values can only be performed through experimental tests on an engine dynamometer using an aircraft engine. It is not possible to fully reflect all changes in the engine’s operating parameters, including mainly the cyclical changes in the stereometry of the shape of the working surfaces as a result of the stochastic wear of the mating planes of the kinematic pairs in the simulation model.

The development trends in aviation internal combustion piston engines for the propulsion of ultralight airplanes and drones result in the constant need to increase the power of the propeller drive system. The authors in [3] discuss the theory of vibrations and the construction of propellers with minimal induced losses. The theory of minimum energy loss propellers depends on consideration of the regular helicoid escaping vortex layer. In [4,5], the dynamics of vortex layers and the consequences of their instability are discussed; it takes place in the case of aerodynamic phenomena of the propeller. The phenomena of angular changes in the position of the rings in the piston grooves as a result of vibrations from the propeller and the nature of the combustion process lead to changes in the oil film distribution [6,7,8,9].

Currently, in order to limit the effects of these undesirable phenomena, many manufacturers are working on the development of synthetic engine oils and oil additives that reduce the friction and vibration of piston rings [10,11,12,13]. Reducing the dynamic viscosity of the oil reduces the internal friction resistance in the oil film, as does the use of many different oil additives reducing the friction in direct contact with micro-inequalities [14,15,16,17]. There are many different techniques that are used to reduce engine drag torque, especially when texturing the cylinder-bearing surface [18,19,20]. Currently, numerous works focus on the influence of the texturing geometry and the structure of oil-bearing micropores on friction losses [21,22,23]. The key parameter determining changes in the moment of resistance to movement of the engine during its operation is mainly the roughness value of the mating surfaces, the pressure values in the space behind the ring, the shape of the sliding surfaces, mutual distances between the piston rings in the piston grooves, the shape of the piston side surface, material parameters of all movable engine units, including ring resilience and gas pressure in the combustion chamber and behind the rings, and local dynamic oil viscosity [24,25]. This makes the selection of materials for the working surfaces of the piston rings of aviation internal combustion engines more and more problematic.

Moreover, in [26,27,28], an attempt was made to verify the degree of dust in the air and filtration to the degree of wear of the main piston mechanisms of internal combustion engines; this is very important for the operation of aircraft engines and their operational safety, and an important aspect of the interaction between the working surface life of the piston rings and the cylinder bore in internal combustion piston engines in aircrafts. Modeling of the oil film in internal combustion piston engines is described in [29,30]. However, no publication has so far considered such detailed working conditions. This topic is ignored by many researchers who model piston engines. Aircraft engines also operate at very low external temperatures; hence, there is a justified need to test the various anti-wear coatings and geometries of the working surfaces of piston rings in order to develop certain trends in the selection of these components. Forecasting the total power loss due to engine friction on the basis of detailed component models is presented in [31].

The influence of selected coatings on friction losses and tribological parameters used on piston rings are presented in [32]. In these studies, in order to increase the tribological efficiency of the piston ring–cylinder pair in internal combustion engines, the piston rings were covered with a Ni–P–TiN coating prepared using electroless coating technology. The research showed a positive effect of the application of TiN-based coatings. Research on anti-wear coatings applied to piston rings was also conducted in [33]. Very promising results have been obtained with various titanium-based coatings. It has been found that the coating should consist of two layers: an abrasion-resistant inner layer made of titanium nitride (TiN) and a lapping-resistant outer layer made of titanium. Applying such a coating resulted in a reduction in wear by up to 40% compared to rings with electroplated chrome coatings. The high wear resistance and low friction of TiN coatings deposited via plasma in the non-lubricated state and at high loads is presented in [34,35,36,37,38]. Reduced friction and wear as well as no tendency to galling have also been proven for titanium suboxide coatings [39,40]. A study of the corrosion and fatigue wear of CrN-coated piston rings is reported in [41]. Surface and interface characteristics confirmed that fatigue wear caused cracking and chipping of the CrN coating. Hence, it is required to introduce ceramic additives to Cr coatings [42]. The comparison of scuffing behavior and wear resistance of potential engineering coatings to automotive piston rings was analyzed in [43]. Integration of traditional ceramic coatings with a hard, low-friction surface has been shown to work well for anti-wear coatings, and amorphous carbon has been shown to improve the frictional strength of the chrome surface. In [44], it was shown that a diamond-like carbon (DLC) coating shows smaller wear and friction under operating conditions similar to those of uncoated piston rings. Thus, it has been proven that the use of a DLC coating contributes to an increase in the service life of the piston elements in internal combustion engines. The tribological behavior of the DLC carbon coating in applications with the use of piston rings is also presented in [45]. The tribological characteristics and the interaction of the surface between the piston rings of the coating and the mixture of energy-saving oils and ethanol fuels with the use of DLC, CrN and TiN are presented in [46]. In this work, the ring coatings include thermally sprayed CrN and physically vapor-deposited (PVD) (DLC). Tribological properties of ring coatings Piston rods were determined using several advanced piston ring coatings and energy-saving lubricants containing friction modifiers. The test results show that the DLC coating causes the lowest wear of the cylinder liner segment and has similar wear as nitrided and CrN-coated piston rings [47,48]. Due to the presence of MoDTC in engine oil, it has been shown that friction and wear are effectively reduced. Adhesive wear is one of the main causes of damage to the mechanical components of engines. In [49], multilayer CrN/DLC/Cr-DLC composite coatings prepared by plasma-assisted chemical vapor deposition (PECVD), a device using the ion plating technique with an unbalanced magnetron in the near field (CFUBMSIP), were characterized. The results show the positive effect of using DLC coatings in mixed structures. The latest research on the measurement of friction losses of aircraft engines has been shared in some of our previous studies [50,51].

The scientific aim of this work is to assess the impact of the application of selected anti-wear coatings, TiN, TiAlN, CrN and DLC1, on changes in the moment of resistance to air movement of the engine on a test stand. The research fills the scientific gap in the field of research on internal combustion piston engines with specialized anti-wear coatings. The constructed test stand using the prototype Vaxell 40i engines (Świątek factory, Poland) reflects the operating conditions of the engine operating in the aircraft. The main advantage of these tests is the assessment of the impact of changes in the dynamic viscosity of the oil with the simultaneous application of anti-wear coatings on the distribution of the characteristics of the average moment of resistance to motion of the combustion engine. This allows for a significant assessment of the behavior of the selected coatings in a running engine. Most of this type of research is based on measurements of the friction coefficients for the selected materials using tribometers, which does not reflect the actual changes in the dynamic and thermal loads on the selected materials, nor the structural elements of the engine. These parameters significantly affect the course of wear, the distribution of friction losses of the selected surfaces of the mating kinematic pairs, and changes in the shape of the piston rings and the piston. As is well known, a change in the shape of the sliding surface of the piston rings has a very significant effect on the oil film thickness distribution throughout the engine operating cycle, on oil consumption, and on the ratio of mixed friction to fluid friction. Bench tests do not allow for precise determination of these parameters but they do take into account a set of all the phenomena, the impact of which is the initial parameter in the form of changes in the moment of resistance to motion of the engine.

## 2. Materials and Methods

### 2.1. Initial Verification Tests of Rings with Anti-Wear Coatings

As part of the preparatory and technological works, tests of the piston rings were carried out, which included measurements of their stereometric shape in all planes cooperating with the piston and cylinder surface, the values of elasticity of the rings, clearance in the ring lock and qualitative tests of adhesion of the coatings to the substrate. Additionally, a measurement of the adhesion of the sliding surfaces of the piston rings to the cylinder surface was carried out using the gap method with the use of a reference cylinder liner and an external light source. The remaining tests included surface roughness measurement for selected anti-wear coatings, and measurement of the piston geometry and the cylinder sleeve surface. All verification tests were carried out before the run-in period and after the run-in period to evaluate the changes in the geometry of the working surfaces of the rings and to evaluate the material properties of the applied anti-wear coatings using an SEM electron microscope and analysis spectroscopic EDS. Metallographic examinations were carried out on transverse sections of the samples using an OPTA-TECH 40 optical microscope (Poland) with digital image recording and a Tescan Vega 5135 scanning electron microscope (Czech Republic). Point analysis of the chemical composition and concentration of the elements was performed on a PGT Avalon X-ray microanalyzer (Czech Republic) on the transverse sections of the samples.

In order to assess the wear of the mating surfaces of the selected kinematic pairs, prophylactic measurements of the thickness of the anti-wear coatings and their adherence to the substrate were made after the tests on the engine dynamometer. In order to implement the selected assumptions for experimental tests, a Vaxell 40i aircraft engine with modified auxiliary equipment was equipped with a package of sealing rings with a modified shape of the sliding surfaces made of ADI thermally modified cast iron with 4 anti-wear coatings, namely, TiN, TiAlN, CrN and DLC_1_. The same coating was applied simultaneously to the upper and lower sealing rings. The tests assumed an asymmetrical shape of the upper sealing ring (the apex of the asymmetry of the rings is shifted towards the crankcase by 20% from the axis of symmetry) and a symmetrical shape of the lower sealing ring. The scraper was the standard shape used by the engine manufacturer. On the plaster the cylinder uses a standard chrome coating with increased surface hardness. The remaining coated rings were made of ADI (Austempered Ductile Iron) cast iron, with a bainitic-austenitic matrix with spherical graphite. The obtained coatings, regardless of the application technology and chemical composition, are characterized by a good bond with the substrate. No delamination of the coatings was observed. The samples of the transverse sections were randomly selected and randomly cut with respect to the circumference of the ring.

### 2.2. Measurements of the Shape of the Sliding Surfaces of Sealing Rings with a Profilograph and Devices for Measuring Selected Working Planes

In order to evaluate the change in the geometry of the sliding surfaces of the sealing rings and the scraper ring, their shape stereometry was measured using the TOPO 01K version 2D profilograph (Poland), calibrated according to the PW-08-L3 procedure (Poland). The measurement uncertainty values are extended uncertainties with a confidence level of about 95% and a coverage factor k = 2. All the measurements carried out had an accuracy of ±0.001 mm. The measurement of the stereometry of the working surfaces of the sealing rings is the basis for assessing the causes of changes in the torque of the resistance to motion of the engine.

As part of the selection of rings from the production batch, 10 rings were selected for each of the TiN, TiAlN, CrN and DLC1 coatings. Then, on the basis of profilograph measurements, pairs of rings (lower and upper) with very similar shapes of sliding surfaces cooperating with the cylinder surface were selected. The deviation in the shape of asymmetry and symmetry of the sealing rings at the height of the barrel outline did not exceed 2 m, and the shift from the axis of symmetry and asymmetry for a given pair did not exceed 20 μm. The error in manufacturing the piston rings did not exceed 10 µm. Such deviations in the dimensions of the shape stereometry working planes does not significantly affect the differentiation of the unit pressure distribution of the sliding surfaces of the rings on the cylinder bearing surface for given pairs of rings mounted in the engine. These dimensions, within these tolerances, were maintained for all anti-wear coatings. The micropore shape topography for the stereometry of the sliding surfaces is adequate for the given texture of the anti-wear coatings. The seal ring sliding surface shape was measured at 10 different randomly selected locations around the circumference of each piston ring after fabrication, coating, profiling with a profile grinder, running-in period, and engine test bench tests.

Additionally, the tangential force was measured for all piston rings. This test for the sealing rings and scraper was performed using special analog-digital instruments. The measurement of the tangential force is used to determine the average unit pressure of the piston ring on the cylinder surface. On the basis of the performed measurements, it is assumed that the average value of the tangential force closing the ring lock for the upper sealing ring installed in the engine for all tests and sets was from 12.11 to 12.56 N. For the lower sealing ring, the tangential force closing the ring was from 12.15 to 12.34 N. In the case of scraper rings, the average value of the tangential force for both cylinders was approximately 26.78 N. During the experimental tests, the rings were selected to have a similar value of the tangential force and a very similar shape of the working surfaces. The clearance was also measured for buckle: for the upper rings, the arithmetic mean of the slide play was 0.32 mm, and for the lower sealing rings, 0.42 mm. The scraper rings had an average play of 0.25 mm.

Only identical values of the control parameters of the piston rings make it possible to determine the real impact of the application of selected coatings on the changes in the moment of resistance to air motion of the internal combustion engine on the test stand. According to internal quality control standards, in ring manufacturing companies, it is assumed that the permissible difference of tangential force for the sealing rings and scraper ring in a production batch should not be greater than 0.50 N, and the value of clearance in the lock should not be greater than 0.05 mm.

### 2.3. The Test Stand for Measuring the Moment of Resistance to Movement of the Main Engine Mechanisms

All experimental tests with the use of various anti-wear coatings were carried out on the prototype Vaxell 40i engine intended for the propulsion of ultralight tourist aircraft. The auxiliary equipment of the engine on the dynamometer was modified in such a way that it was possible to obtain operating conditions similar to the actual operating conditions of the engine for flying ultralight aircraft. The stand was designed so that it is possible to assess with the greatest possible accuracy the changes in the moment of resistance to the movement of the piston–piston rings–cylinder unit, as well as the shaft and main and crankshaft bearings. The engine was deprived of a timing system and any additional auxiliary devices that could create additional resistance to engine movement. The moving parts were driven by an electric motor by means of an articulated gearbox with a torque meter, such as the crankshaft, connecting rods, piston and piston rings. The timing system was a stationary part. The engine heads were reworked to obtain additional compressed air pressure to simulate the combustion process. Of course, the real conditions and the course of gas forces in the engine’s work cycle cannot be perfectly reproduced, but within the framework of the project it was possible to obtain a maximum pressure of the working medium at the level of 20 bar. Such an air pressure was a reference point on the test stand. The Eurotherm 3504 temperature controller (Faraday Close, Worthing, UK) was installed on the test stand, which allows for precise regulation of the temperature of the oil and cooling liquid. The KD485 communication converter (Faraday Close, Worthing, UK) was introduced, which is recommended by the manufacturer to fit the EIA 485. The station was equipped with a series of thermoelectric sensors that determined the temperature of the oil and coolant to an accuracy of ±0.1 °C. The combination of such elements with the PID controller allows to maintain the set engine oil temperature while measuring the moment of resistance to engine motion, even at temperatures above 100 °C. This enables the determination of the characteristics of changes in the torque of the resistance to motion of the engine on the dynamometer for the set of rotational speeds of the engine at a specific temperature of oil and cooling liquid. Another disturbance that may affect the reliability of the measurement results are the deviations in the measurement values caused by the too-short running-in period of the individual test ring packages for the given coatings. It should be noted that this time is highly dependent on the surface topography and the surface hardness of the coating applied to the piston rings.

### 2.4. Measurements of Dynamic Viscosity of the Engine Oil Intended for Testing

**Figure 1:**
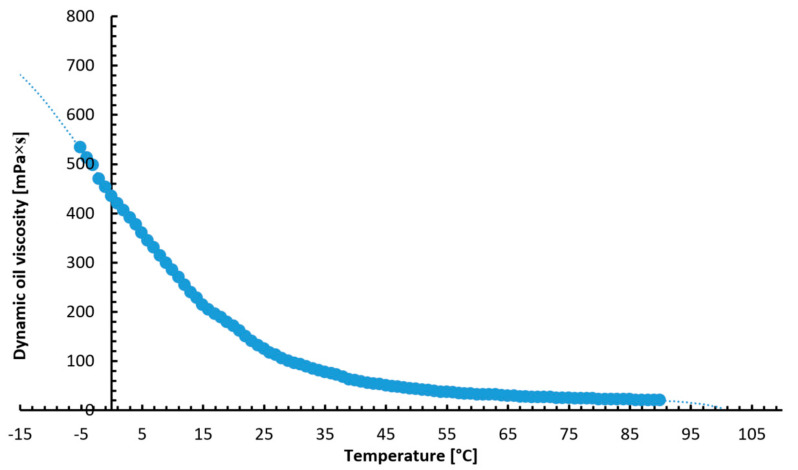
Dynamic viscosity of the 5W-30 oil in the temperature range from −5 to 100 °C.

5W/30 engine oil was used for the bench tests. The dynamic viscosity of the oil was measured with a Bookfield DV-II + Pro rotational viscometer (Harlow, Essex, UK) running with the Rheocalc32 or Wingather programs (3.3.49) (Middleboro, MA, USA). The characteristics of the test oil viscosity changes as a function of temperature, as shown in Figure 1. During the test tests, the oil was periodically changed. Despite the fact that the engine oil during the research tests was not significantly consumed due to frictional resistance, and as a result of the combustion process (there is no combustion process of the air–fuel mixture on the test bench), the course of the wear process of the anti-wear coatings could cause slight contamination of the engine oil. Therefore, after the break-in period of the rings, the oil was replaced with the same production batch from the same container. It ensured a very high reliability of the obtained results of the measurement of the resistance to motion of the engine. The temperature characteristics of the dynamic viscosity of the 5W/30 oil is shown in Figure 1.

### 2.5. The Measurement of the Moment of Resistance to Movement of the Piston–Piston Rings–Cylinder Mechanism on the Test Stand

**Figure 2:**
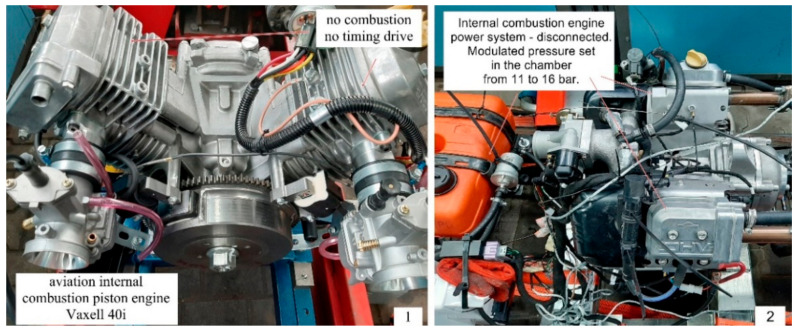
The view of the test stand and the engine torque sensor mounted on it.

The KTR Dataflex torque meter (KTR Systems GmbH, D-48432 Rheine, Poland) was used to measure the resistance torque of the piston–piston-rings–cylinder mechanism on the test stand with the Vaxell 40 engine with a modified main and auxiliary mechanism (Figure 2). The torque meter is characterized by high accuracy in measuring small moments of resistance to movement of an internal combustion engine. The electronic system was built into the housing, which reduces the risk of signal distortion caused by external factors. The measurement is performed at a sampling frequency of 16 kHz. The Dataflex is terminated with analog outputs that provide a 0–10 V voltage signal and a 4–20 mA current signal. The measurement of the rotational speed of the drive shaft is performed with an accuracy of 60 pulses per revolution. The linearity error including hysteresis was less than ±0.5%. The influence of temperature on the measurement result was 0.5%/10 K. The measurements were carried out at the same ambient temperature of the engine compartment, i.e., 23 °C. The inaccuracy of the output voltage for speed measurements was DC ± 0.2%. An intermediate connection terminal DF2 was also used, which was mounted on an O-type DIN rail. The torque signal was filtered on five levels so that the torque spikes were reduced while the signal was on indicated or registered. The F/U converter was integrated with the impulse converter providing a DC voltage signal with a value in the range from 0 to 10 V. A set of Radex-Nc clutches was used to neutralize the undesirable vibrations between the electric motor controlled by the inverter and the internal combustion engine.

The stand ensures the accuracy of the regulation and measurement of the rotational speed of the engine crankshaft at the level of ±1.0 rpm. Based on the average of 5 measurements of the engine rotational speed, temperature, oil viscosity and geometric dimensions of the engine, the mean values of the moment of resistance to motion were obtained, which were the basis for the results of the primary measurements. The maximum standard deviation of the measurement results did not exceed ±1.0%. The data of the aircraft engine on the dynamometer are presented in Table 1.

### 2.6. Method of Testing for Coatings Used in the Engine Tests

Both some new materials for coatings and surface treatment techniques are needed to meet the new requirements of aircraft piston engines, in particular those operating in conditions of a high mixed friction share. Data concerning the coatings used in the engine dynamometer tests, which were applied to the sliding surfaces of the piston rings, are presented below. The hardness and Young’s modulus of the coatings were measured using a depth-sensing indentation (DSI) technique (NanoTest 600 instrument from Berkovich indenter, Micro Materials Limited. C, CIC Cambridge, MA, USA). The surface was measured using the same gauge tip profile (T8000, Hommelwerke GmbH, Schwenningen, Germany) after the run-in period at the moment of stabilization of the torque value during several measurements under the same engine operating conditions. In these experimental measurements, the nanoindentation loading rate was 10 nm/s, and the hardness and elastic modulus values were identified on the basis of average values of the load displacement between 1/7 and 1/10 the film thickness. In addition, an interferometric non-contact optical profilometer and microscope (MicroXAM^®^, Universal Way, Tucson, AZ, USA) were used for measuring the parameters of roughness, finish and texture. Scanning tests were performed in the probe microscopy (SPM) mode dedicated for surface topography imaging.

The tests on a macro scale were carried out using a device for testing the ball wear on a target (ITeE T-01M tester, Radom, Poland). The ball-on-dial configuration is the most popular system for assessing the tribological properties of materials in direct contact and is a reference point for tests carried out on an engine dynamometer. The behavior of the TiN, TiAlN, CrN and DLC1 coatings was carried out in contact with the 100Cr6 steel ball. The test was carried out for the following parameters: load *p* = 10 N; sliding speed: v = 0.1 m/s; frictional displacement: s = 1000 m; relative humidity: 55 ± 5%; and ambient temperature 22–25 °C. Microscale friction measurements were made with a T-23 ball tester (Radom, Poland). The upper samples destined to have contact with the test coatings were spikes with a diameter of d = 5 mm made of steel (100Cr6) and ceramics (zirconium dioxide stabilized with yttrium oxide Zr_2_O_3_∙Y_2_O_3_), which moved at a certain sliding speed along a specific path. The first measurement was made with a load of 50 mN. Then, the measurements were carried out for every increase of 10 mN until reaching a final value of 100 mN. The lower sample was measured in duplicate for each load change. In the tibometric tests, the distance between the frictional scratches caused by the movement of the ball on the samples against a flat surface was 0.2 mm. The measurement was performed in ambient temperature and at a speed of 25 mm/min and a displacement of 5 mm (up and down). For all research cases, the ambient humidity in the laboratory was 45 to 50%.

## 3. Results and Discussion

### Properties of the Coatings Used in Tests with an Engine Dynamometer

Prior to deposition of the rings, they were mirror polished and ultrasonically cleaned in acetone and alcohol gradually, each ring for 15 min and dried for approximately 20 min in a vacuum oven. The DLC1 coating was applied with PVD technique by ion sputtering at a temperature of <250 °C. Therefore, the imposed layer is suitable for coating ADI piston rings made of ductile iron. It does not change the resilience properties of the piston rings. Coatings’ carbon bonds are often a mixture of graphite and diamond bonds. A-C: H coatings are known as inert coatings with a low surface energy, thus being perfect for covering moving parts. The addition of metal (W or Ti) or non-metallic (F or Si) atoms to the carbon matrix changes the chemical and mechanical properties of the aC:H coatings.

The TiN and CrN coatings were obtained in PVD processes: TiN by arc evaporation. Prior to the deposition of the TiN coating, purification of the Ti ion bombardment was performed to improve the adhesion. Arc evaporation of the TiN was performed at a temperature of 500 °C. A CrN coating was applied by a PVD process by ion sputtering at a temperature of <270 °C. To achieve the full benefits of the TiAlN coatings, good interfacial adhesion between the imposed layer and substrate is necessary. The TiAlN coating was deposited using the same method as CrN at a temperature of 200 °C. The DC substrate bias voltage ranged from −40 V to −150 V, and the flow of Ar and N2 was independently controlled by the mass flow controller. The average deposition time of all the coatings was approximately 60 min. The distance of the source from the prepared substrate was approximately 200 mm. The power used was 2.5 kW and the gas pressure was between 1 and 1.5 Pa. The cathode current was 60 A. Coating the surface is an effective method of improvement durability of the materials used in aggressive environments, especially in the production of piston rings. By selecting the appropriate PVD coating methods and coating materials for the substrate material steel or ductile iron, you can extend the total service life of the substrate material and increase the time between heavy repair of the internal combustion engine.

The average thickness of the applied coating on the circumference of the a-C:H ring was 1.53 ± 0.15 μm and the average thickness of the Ti intermediate layer was 0.31 ± 0.04 μm. Coating hardness, measured at the surface of the piston ring, was 21.85 ± 0.45 GPa. In contrast, the modulus of elasticity was 156.36 ± 2.34 GPa. The TiAlN coating applied to the piston rings after the running-in period had an average thickness of 2.98 × 0.35 μm, a hardness of 36.21 ± 2.22 GPa and a Young’s modulus of 291.23 ± 11.21 GPa. The TiN surface has similar characteristics to TiAlN and CrN because it was layered using a similar technique. However, the size and density of the macroparticles of TiN are smaller than that of the other two coatings. The TiN coating after the running-in period was characterized by an average thickness measured on the circumference of the ring of 4.82 ± 0.46 μm, a hardness of 29.56 ± 1.82 GPa and a Young’s modulus of 347.45 ± 12.23 GPa. Similarly, the CrN coating had an average thickness of 4.34 ± 0.43 μm, a hardness of 24.99 ± 2.34 GPa, and a Young’s modulus of 288.76 ± 16.23 GPa.

The slight thickness of the coatings used to layer surfaces of the piston rings could indicate that the wear of the rings would be complete if they were naturally run-in in the engine. This is not the case due to the high hardness of all applied coatings. The coatings were selected in such a way that it was possible to evaluate their impact on the microtexture obtained after the running-in period without major changes in the macrocontroller of the sliding surfaces of the piston rings. In the case of a change in the macrometric shape at the micrometer level, it would be difficult to conclude what was the cause of the changes in the value of the torque of the resistance to motion of the motor. It is the macrospheres of the rings that largely influences the oil film distribution under fluid friction conditions. The share of mixed friction occurs only in the initial periods of the engine expansion stroke, when the increase in operating pressure in the combustion chamber is the highest possible. This process takes place over a period of approximately 30 degrees of crankshaft rotation, where the entire engine cycle covers 720 degrees. However, despite such a small share of mixed friction, many researchers indicate that the values of these torque increments in the period of direct contact of opposite shells may be significant. Therefore, an attempt was made to assess the scale of this phenomenon to the conditions of an engine dynamometer. As the research shows, despite the constant shapes of the rings at the macro scale, a small share of mixed friction may cause a complete change in the torque value in the entire range of the considered engine rotational speeds. To a large extent, the nature of the distribution of values over the entire velocity cycle does not change much because it largely depends on the macro-shape and fluid friction. It can be said unequivocally that the hardness of the coatings alone does not say much, apart from the degree of wear of the coatings after the run-in period in the degree of macro-shape, e.g., changes in the macro-shape of the symmetry outline or asymmetry of the ring shape at the level of 1 to 2 micrometers. However, hardness largely affects the distribution of micro-inequalities, the texture of which may be of importance in the case of mixed friction conditions. In this case, due to the current design trend of motors, the aim is to obtain the lowest possible surface roughness. This is due to the low thickness of the oil film between the cylinder running surface and the upper sealing ring. Nevertheless, in the case of very low roughness, as in the case of DLC1 coatings, a trend of increasing the torque can be observed in relation to the TiAlN coating. Perhaps in this case, the texture of this surface serves as an oil reservoir and the load-bearing capacity of the oil increases, which additionally supports the ring to initiate fluid friction conditions. In the case of other coatings, the micro-inequalities are so large, it is likely that the micro-peaks of the unevenness meet and an additional increase in the moment resulting from the interaction of two materials.

Although the initial surface roughness of the substrates was the same, the resulting roughness after coating was significantly different. The highest surface roughness had TiN (115 ± 9 nm RMS), then CrN (75 ± 4 nm RMS), then TiAlN (59 ± 3 nm RMS) and DLC1 (31 ± 2 nm RMS). The high roughness of the TiN coating may result from the nature of the substrate layer. Before applying the coating layer, the rings, apart from the shape treatment, were also subjected to a polishing process. This process did not change the cross-section and the basic shape to such an extent that it significantly influenced the result of the measurement of the moment of resistance to motion of the engine.

**Figure 3:**
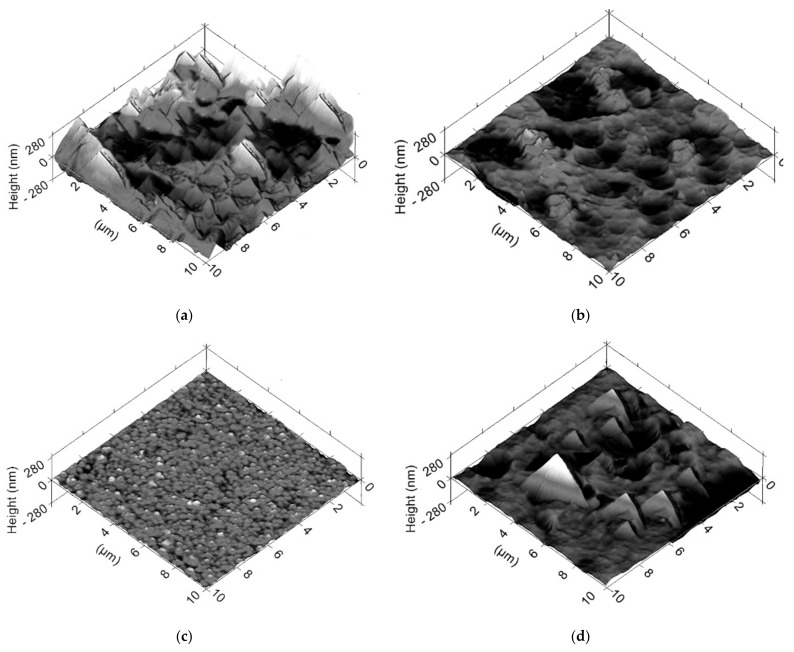
3D topographic images of the SPM coatings applied to the sliding surfaces of the piston rings after the application and running-in period: (**a**) TiN, (**b**) CrN, (**c**) DLC1 and (**d**) TiAlN.

The corresponding three-dimensional topographic images of the samples are shown in Figure 3. The RMS surface roughness after the run-in period following the TiN, TiAlN, DLC1 and CrN coatings is given above. TiN had the highest surface roughness, then CrN, TiAlN and DLC1. The topographic images show the presence of high and sharp peaks of the micro-inequalities together with deep valleys on the surface of the TiN sample (Figure 3a). In fact, the characteristics of the TiN sample are so irregular that they essentially represent the Berkovich tip used for SPM scanning. The result is imaging of the pyramidal defects (Figure 3a). In the case of CrN, a different surface roughness morphology can be seen. The peaks are not that high or sharp. Nevertheless, the CrN coating contains a significant proportion of pits in its topography (Figure 3b). The surface of the DLC1 sample is practically smooth, with no major surface defects (Figure 3c). The surface roughness of the DLC1 coating is practically very much small and evenly distributed over the surface. The TiAlN sample shows some deep defects and slight peaks of micro-inequalities similar to those in the case of TiN and CrN coatings; however, the scale of these irregularities and dimensions are significantly smaller. The density of the micro-inequalities for the TiAlN coatings is higher than for the other TiN and CrN coatings due to smaller stereometric dimensions (Figure 3d). Probably, since the roughness of the TiN and CrN coating was high, the energy required to produce plastic deformation under mixed friction conditions was also high, which resulted in high friction. Ceramic and carbon coatings, such as titanium nitride (TiN) and diamond-like carbon coatings (DLC1), have good mechanical properties and chemical inertia, but under mixed friction conditions, the given surface textures have a different friction distribution nature.

**Figure 4:**
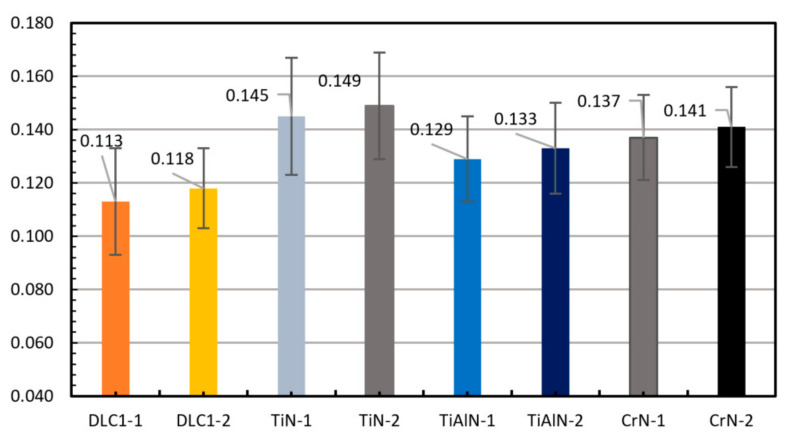
Average values of the coefficients of friction measured for test specimens with the same coatings as on the piston rings.

Figure 4 shows the values of the friction coefficients obtained with the T-23 microtribometer (Radom, Poland). For all tested materials, the friction coefficients were lower in the configuration with the 100Cr6 steel ball (1-ball) than in the configuration with the ceramic ball Zr_2_O_3_∙Y_2_O_3_ (2-ball). According to the diagrams, the smallest friction coefficient is characteristic for the coatings DLC1, TiAlN, CrN and TiN. The DLC1 coating is characterized by a lower coefficient of friction, by 22.07%, with the 100Cr6 ball than the TiN coating. For the Zr_2_O_3_∙Y_2_O_3_ ball, the difference for these coatings was 20.80%. In the case of the TiAlN and CrN coatings, the value of the coefficient of friction in relation to the DLC1 coating is successively higher by 12.40% and 17.52% for the 100Cr6 ball and 11.28% and 16.31% for the Zr_2_O_3_∙Y_2_O_3_ ball. This explains the initial increased resistance to motion detected on the test stand. In comparison with the microstructure, it can be concluded that where the share of mixed friction is greater, it translates into the direct contact between of the ring coatings and the cylinder surface.

The macroscale tribological properties were analyzed with a T-01 M ball tester (Radom, Poland) in dry sliding friction conditions and in an oil environment of 5W/30. The sample surface was wetted with the oil. For this purpose, the coatings applied to the previously prepared samples with dimensions allowing the test to be carried out were used. The samples had the same properties as the coatings applied to the piston rings. A 100Cr6 steel ball was selected as a counter sample. The wetting consisted of applying an oil mist to the sample with the coating applied, and two sprays of the liquid were made to obtain the same thickness of the oil.

**Figure 5:**
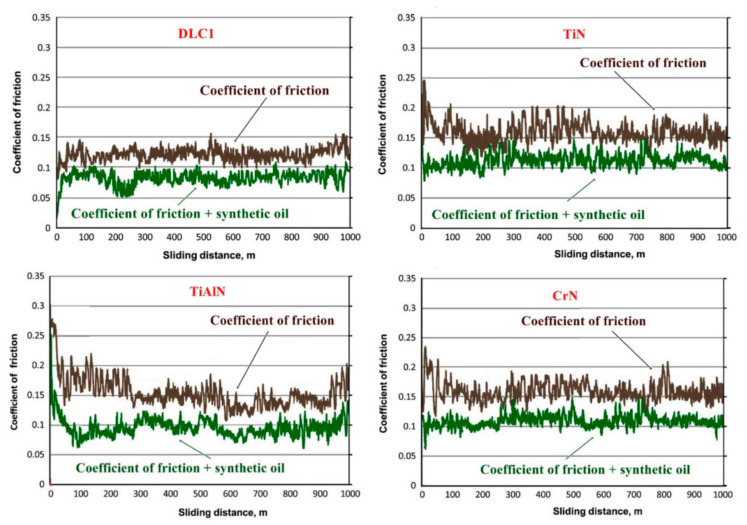
Coefficients of friction and wear recorded in dry friction conditions for the 100Cr6 steel ball system: DLC1 coating, TiN coating, TiAlN coating and CrN coating.

Figure 5 shows the tribological characteristics recorded for the four anti-wear coatings applied to specially prepared samples of the same nature as in the case of rings. The base material was thermally modified ductile iron. The thickness of the coatings and all their parameters are the same as in the case of coatings applied directly to the rings. The coatings were applied in the same conditions from one technological batch. The results show the change in the value of the friction coefficient in the test cycle for the DLC1, TiN, TiAlN and CrN coatings. The materials were tested under a constant speed and load.

The lowest value of the coefficient of friction was achieved by the DLC1 coating, which is the key parameter determining changes in the value of the engine resistance moment on the engine dynamometer. The value of the friction coefficient in the initial period of dry friction was lower; then, it increased to be in the range of about μ ≈ 0.127 along the friction displacement s = 1000 m. The DLC1 coating was wetted with a lubricant with viscosity coefficients corresponding to about 20 °C. Initially, an increase in the value of the friction coefficient from about μ ≈ 0.03 to μ ≈ 0.09 was noted. This value was maintained up to a distance of about s = 200 m, then dropping sharply to the value of about μ ≈ 0.06, and then increased after s = 250 m. This value increased from μ ≈ 0.06 to μ ≈ 0.08–0.09 and remained in this range to the end of the path s = 1000 m. The decreases and increases in the value of μ could be caused by the coating lapping with the test ball material or by stochastic wetting of the surface in this range of the measuring path. It should be assumed that in the longer period of cooperation, the value of the friction coefficient for DLC1 is in the range from μ ≈ 0.08 to 0.09.

In the case of the TiN coating, a large increase in the value of the coefficient of friction μ was observed in the initial period of the measuring path s = 10 m. After this period, the value of μ decreased from μ ≈ 0.24 to μ ≈ 0.15 in the range of the distance s = 170–300 m. After this period the value of μ has increased to μ ≈ 0.18. Along the way s = 600 m, the value of the friction coefficient decreased and stabilized, ranging from μ ≈ 0.14 to 0.16. In the case of wetting the TiN coating with the liquid, the value of μ initially to the path s = 230 m was approximately μ ≈ 0.11. After this period, it increased and was stable in the range of μ ≈ 0.11–0.13.

In the case of the TiAlN coating, the value of the friction coefficient in the initial period was higher than for the TiN coatings and amounted to about μ ≈ 0.27, then decreasing cyclically to the path of about s = 600 m. After this period, the value of μ was approximately μ ≈ 0.13–0.14. Along the measurement path s = 900 m, the value slightly increased to the range of about μ ≈ 0.15–0.16. The TiAlN coating, after wetting with the lubricating liquid, initially had a high μ value, which decreased to the path s = 100. During this period, the value of μ decreased from μ ≈ 0.25 to μ ≈ 0.06. This value was unstable from the distance s = 100 to the distance s = 700 m. The value of μ in this period for the TiAlN coating varies from μ ≈ 0.06 to μ ≈ 0.12. On the way of the test s = 700 m, the value of μ stabilized and amounted to approximately μ ≈ 0.11.

In the initial period of the measuring track in dry friction, the CrN coating had the value of the friction coefficient μ ≈ 0.22; then, it decreased to the value of μ ≈ 0.16–0.17 and stabilized in this range until the end of the measuring track s = 1000 m. The value of μ initially increased in the range s = 0–300 m from μ ≈ 0.10 to μ ≈ 0.12. After this period, the value of μ was stabilized until the end of the measuring path s = 1000 m and stayed in the range of μ ≈ 0.11–0.13.

Based on the tribological test and the exchange analysis of the μ value, it can be concluded that all coatings have a fairly stable μ value course when wetted with liquid. Only in dry friction, the μ values are very different and unstable. Nevertheless, all coatings at the end of the measuring path have a fairly stable μ value even in dry friction. Therefore, it can be concluded that all coatings are perfectly suited for use in internal combustion engines as an anti-wear and friction-reducing layer. DLC1 and TiAlN coatings, which have a low value of the coefficient of friction μ, are the best. Nevertheless, it can be said that the TiN and CrN coatings in the entire scope of the test show a greater stabilization of the friction coefficient value in relation to the TiAlN coating. In this case, it can be stated that these coatings, despite their high μ values, can also be used in piston ring coatings. According to the μ analysis, it can be said that these coatings behave in a similar way in comparison to the engine dynamometer tests. However, it is difficult to explain some deviations in the TiAlN coating. Therefore, it can be said that such changes certainly depend to a large extent on the correlation of the dynamic viscosity of the oil for a given temperature range to the topography of the coating surface. It is largely determined by the proportion of dry, mixed friction to fluid friction. Unfortunately, in conditions of dynamic work, it is more difficult to assess the stochastic nature of these flows. One can only suggest a comparison of the values of tribological tests and material analyses with the results obtained from the measurement with an engine dynamometer.

Figure 5 shows the results of one measurement cycle for five measurement tests with the given engine operation parameters. This value was marked as the measurement error at the value axes in the form of bars. The measurements were taken after the running-in period of the piston rings. The presented charts for the anti-wear coatings TiN, TiAlN, CrN and DLC1 show the distribution of the torque of the resistance to motion of the motor at a temperature of 40 °C. According to the presented figures, it can be seen that the standard deviation of the individual measurement values for the five measurements is very small, which is the basis for the further testing for the remaining engine operating parameters. Such a high accuracy of the obtained results ensures reliable conclusions. It should be mentioned that in the case of tests carried out on a real engine with the use of an engine dynamometer, obtaining such a high accuracy is a very big challenge in relation to the method of control and construction of the test bench and the production of individual components of the main engine of the internal combustion engine. Surface coverings used in the production of piston rings are to meet various requirements, e.g., better running-in of the rings by obtaining a low coefficient of friction after the running-in period or better protection against scuffing under temporary conditions of mixed friction. Such phenomena are difficult to reconcile and are closely related to the hardness of the anti-wear coatings and their behavior after the running-in period. Therefore, all tests on the engine dynamometer were carried out with particular emphasis on changes in the moment of resistance to movement of the engine after the running-in period of the coatings. The summary of the torque gives an overview of the differentiation of friction losses for selected materials over the entire engine operation cycle after the running-in period. In the process of engine development, the aim is always to achieve the highest possible efficiency of these engines and obtain the maximum concentration of power. This leads to a continuous increase in the parameters of the engine’s working processes, i.e., the maximum and average pressure and temperature thermodynamic cycle. Therefore, the interest in the use of ceramic and titanium-doped coatings and coatings with a low thermal conductivity coefficient has increased. These concepts envisage reducing the amount of heat dissipated to the cooling system, which increases the efficiency of the engine. In the case of aero-engines without supercharging, increasing the temperature of the cylinder walls and insulation of the rings reduce the filling of the engine, and the efficiency gains obtained in this combination are small. Such verification is possible thanks to the measurement of the moment of resistance to motion of the motor and thus the assessment of the possibility of improving the mechanical efficiency of the motor.

**Figure 6:**
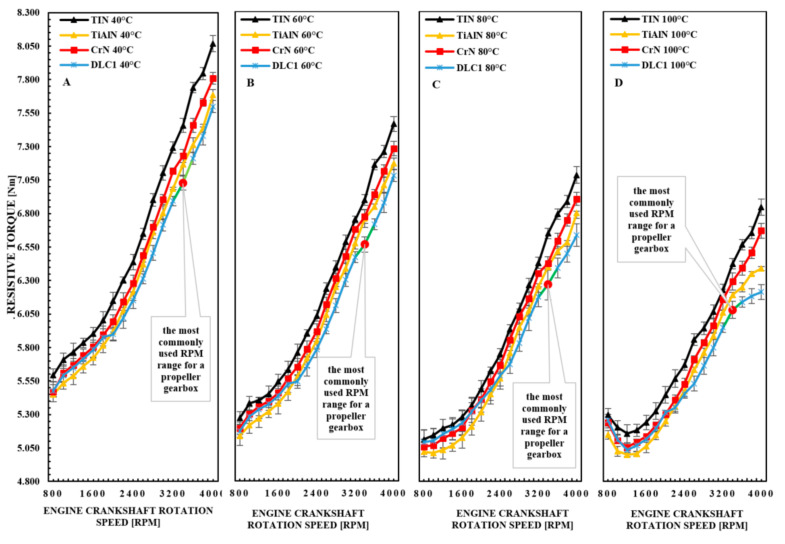
Waveforms of the engine resistance to motion for the TiN (pak1-p1), TiAlN (pak1-p2), CrN (pak1-p3) and DLC1 (pak1-p4) coatings at an oil temperature of (**A**) 40, (**B**) 60 (**C**) 80 and (**D**) 100 °C in the rotational speed range shaft from 800 to 4000 rpm.

Figure 6A shows that the lowest resistance to motor motion at 40 °C is produced by the DLC1 coating compared to other TiN, TiAlN and CrN coatings. The highest resistance to engine motion is generated by the TiN coating in all the considered oil temperatures and the range of rotational speeds of the crankshaft. DLC1 coatings reduce the drag torque of the motor in the most commonly used range of shaft speeds from 3200 to 3600 rpm in the range from 5.431 to 6.877% with respect to TiN coatings. This is due to the properties of the DLC1 coatings: the hardness and roughness of the mating surfaces, which probably translates into smaller shares of mixed friction after GMP in the expansion stroke. This is certainly partly due to the low surface roughness value, which is 31 ± 2 nm RMS for the DLC1 coating. The reduction in the torque of the motor resistance for the DLC1 coating for higher rotational speeds is certainly also related to its lower hardness compared to other CrN, TiN and TiAlN coatings. The rationale for this is that the lapping products from the DLC1 coating have migrated to the opposite face of the cylinder bore. Certainly, these impurities result in better properties for keeping the oil on the cylinder surface. The DLC1 coating also has a lower modulus of elasticity, which may indicate a better performance in lapping mating surfaces. This value is lower by more than 191.09 GPa in relation to the TiN coatings.

In the conditions of operation of thermally highly loaded aircraft engines, it can be expected that the share of mixed friction in liquid friction in the entire engine operation cycle may amount to several percent. The value of this share results mainly from the geometry of the mating surfaces, local dynamic oil viscosity and pressure values in the combustion chamber. In addition, it is determined by the mutual position of the piston rings in the piston grooves, their mutual distance and the distance of the upper sealing ring from the piston crown. The rotational speed of the engine’s crankshaft is a significant factor. On the test stand, the measurement of the engine resistance torque includes the frictional resistance of the piston, piston rings cooperating with the cylinder surface and the frictional resistance of the engine crankshaft and its bearings and connecting rods. The frictional force between the piston guide the surface, and the cylinder running surface depends on the piston material and cylinder running surface and their properties, mainly the surface roughness and the roughness distribution texture. These parameters become particularly important only after the break-in period of the mating kinematic pairs. Therefore, the choice of coating materials is particularly geared towards micro-changes in geometry after the run-in period. It is also an important correlation of the adhesion and cohesion phenomena of engine oil to surface layers of coatings. The most difficult task is to determine, based on the analysis of the experimental results with the use of an internal combustion engine, the friction conditions between the piston and rings and the cylinder surface. They are not stabilized in nature and take place in the conditions of fluid friction and partially mixed friction, or even boundary friction. It can be assumed that the share of increased mixed friction is visible in the form of a sudden increase in the torque of resistance to movement of the engine in the selected range of engine rotational speed at a given oil temperature. However, there is no clear evidence for this fact, but it can be assumed with high probability that the cause of this condition is the increased frictional resistance resulting from breaking the continuity of the oil film between the upper sealing ring and the cylinder bearing surface.

From Figure 6A, it can be seen that the DLC1 coating, in the range of the rotational speed of the crankshaft from 800 to 2000 rpm, is characterized by higher friction losses than the TiAlN coating from 0.494 to 1.058%. The dependence of the increased resistance to motion of the engine in the case of the DLC1 coating in relation to other coatings can be justified by the phenomenon of better adherence of the oil to the DLC1 coating in relation to other coatings at low displacement velocities of the planes of the kinematic pairs. In the case of DLC1 coatings, given their slight roughness and hardness, the direction of these changes should be the opposite. Thus, the only valid justification may be the fact that the DLC1 coatings, despite a slight roughness, help to increase the coverage of the sliding surface of the rings with oil over most of their height. However, this does not mean that there is a high proportion of mixed friction there. This fact excludes the low surface roughness and additional lubricating properties of the particles formed as a result of lapping the surface of the rings and the smoothness of the cylinder. Note that the roughness difference between the DLC1 coating and TiAlN coating is around 28 nm. For the CrN coating, the roughness difference compared to the DLC1 coating is about 44 nm. It should also be indicated that these values may not be identical on the circumference of the ring, but nevertheless they should be considered an average value and adequate for an objective assessment of physical phenomena.

The CrN coating applied to the sealing rings reduces the resistance to motion of the motor from 1.704 to 3.613% in the range of shaft rotational speeds from 800 to 4000 at a temperature of 40 °C in relation to the TiN coating. This is mainly due to the lower surface roughness after the run-in period of the CrN coating compared to the TiN coating (the difference is about 40 nm RMS). This is greater than the surface roughness of DLC1, which is 31 ± 2 nm RMS.

Similar changes in the characteristics of the motor resistance torque for the TiN, TiAlN, CrN and DLC1 coatings can be seen in Figure 6B,C. In Figure 6B, representing the engine operating conditions at an oil temperature of 60 °C, we can see an approximation of the value of the generated torque of resistance to movement of the engine for the CrN and DLC1 coatings. The difference in the values of this parameter is from 0.394 to 0.409% in the range of rotational speeds of the crankshaft from 800 to 1800 rpm. In this speed range, the DLC1 coating shows lower friction losses. However, at this temperature it shows greater friction losses than the TiAlN coating. In the range of the nominal engine speed from 3200 to 3600 rpm at a temperature of 60 °C, the greatest resistance to motor movement is generated by the TiN coating, followed by TiAlN, CrN and DLC1. A similar trend in changes in the value of the engine resistance torque is observed for oil temperatures of 80 and 100 °C (Figure 6C,D). For an oil temperature of 80 °C (Figure 6C), the difference between the values of the torque of resistance to motion in the range from 3200 to 3600 rpm for the TiN and TiAlN coatings is from 2.731 to 4.091%, and for the combination of TiN and DLC1 coatings from 4.277 to 6.233%. In the case of the measurement group for an oil temperature of 100 °C (Figure 6D), the difference in the value of the motor drag torque is as follows: for the TiN and TiAlN coatings it is from 2.417 to 4.540%, and for the TiN and DLC1 coatings from 4.174 to 6.512%. Waveforms of the engine resistance torque for an oil temperature of 100 °C significantly differ from other test series for lower oil temperatures. In Figure 6D, it can be seen that all TiN, TiAlN, CrN and DLC1 coatings in the speed range rotational speed of the crankshaft from 800 to 1000 rpm show an increasing tendency in the moment of resistance to motion in relation to the rotational speed of the shaft in the range from 1200 to 1400 rpm. It is probably caused by the presence of a large share of mixed friction in relation to liquid friction in this range of shaft rotational speeds.

This means that the surface roughness of the coatings applied to the piston rings and the opposite surface roughness are greater than the thickness of the oil produced between these elements. This is mainly related to the sharp decrease in the dynamic viscosity of the oil. Nevertheless, it can be assumed that the DLC1 and TiAlN coatings turned out to be very effective in counteracting this phenomenon. As a result, the continuity of the oil film is broken and the torque of engine resistance increases. Nevertheless, the waveforms of the moments of resistance to movement of the engine for an oil temperature of 100 °C are similar to the waveforms for the temperature of 40, 60 and 80 °C. In the final area of the engine operation in the range of crankshaft rotational speeds from 3600 to 4000 rpm, an increase in the differences in the values of the resistance to motion of the engine for the TiN, TiAlN, CrN and DLC1 coatings can be observed. In this case, the difference in the value of the engine resistance torque for the TiN and DLC1 coatings for the rotational speeds of the crankshaft in the range of 3800–4000 rpm is from 6.817 to 9.102%.

From Figure 6A–D, it can be seen that the lowest resistance to movement of the engine in the range from 800 to 2000 rpm, regardless of the oil temperature, is produced by the TiAlN coating. Based on the analysis of the characteristics of the moments of resistance to motion of the engine for the acquired temperatures, it can be said that the DLC1 coating contributes to the greatest reduction in engine friction losses in the range of shaft rotational speeds above 2000 rpm. The particular properties of TiAlN in reducing mixed and internal friction losses in the oil film in the low engine speed range can be attributed to its low roughness, which equaled 59 ± 3 nm RMS, and its high hardness of 36.21 ± 2.22 GPa. Theoretically, the DLC1 coating should have more favorable parameters related to the reduction in friction losses. Nevertheless, such relationships arousing great interest were noted. Probably an advantage of the TiAlN coating is also the advantageous arrangement of the micropore texture for this set of mechanical elements. Therefore, it can be argued that they have an additional oil-absorbing function, but to such an extent that they do not significantly increase the increase in the resistance to motion resulting from the internal friction in the oil film. On the other hand, the micro-inequalities are so small that there is not a large share of mixed friction in the entire engine operating cycle. In order to explain this phenomenon in detail, it would be advisable to make a position that allows to define the oil wettability of the surfaces concerned, in particular the sliding rings, which would be extremely difficult with an engine running at high rotational speed.

On the basis of the experimental tests of the engine resistance torque, it was found that the friction share of the piston rings for the engine on the test stand was approximately 45% of the friction losses of the entire engine. This value was established on the basis of additional research. In the engine on the stand, the measurement of the torque with and without piston rings was carried out (the differences reached approx. 45%). Assuming this dependence, it can be calculated that for the most frequently used shaft rotational speed in the range from 3200 to 3600 rpm, the DLC1 coating causes an approximately 14.471% reduction in friction losses of the piston rings in relation to the TiN coatings.

**Figure 7:**
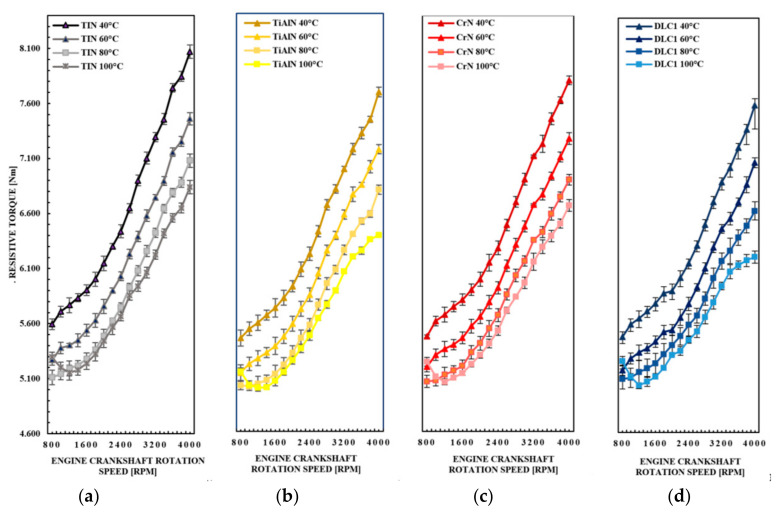
The waveforms of the torque of the resistance to motion of the motor for the (**a**) TiN, (**b**) TiAl, (**c**) CrN and (**d**) DLC1 coating in the range from 800 to 4000 rpm.

Figure 7a–d shows that for the TiN, TiAlN, CrN, and DLC1 coatings, a very similar character of the resistance torque waveforms in the entire engine speed range is observed. The greatest deviations are observed for the oil temperatures of 80 and 100 °C, where the dynamic viscosity of the oil is significantly reduced. As is well known, this leads to a reduction in the thickness of the oil film between the piston rings and the cylinder running surface. In the figures below, for all coatings in the range of shaft speeds 800 to 2000 rpm, slight differences in the values of the moments of resistance to motion of the engine for temperatures of 80 and 100 °C are observed. The greatest differences in the value of this parameter are observed for the DLC1 shell. For all coatings for the shaft rotational speed of 800 rpm, a significant increase in the value of the torque of resistance to movement of the engine at 100 °C, exceeding the value of this parameter even for temperatures of 60 and 80 °C, is observed. The biggest increases in value moments of resistance to motion for a temperature of 100 °C for a shaft rotational speed of 800 rpm are observed for the CrN and DLC1 coatings.

## 4. Conclusions

Based on experimental studies with the use of an aviation internal combustion engine with the use of TiN, TiAlN, CrN, DLC1 coatings on the sliding surfaces of the sealing rings, the following conclusions can be drawn:

(1) DLC1 coatings, in relation to other anti-wear coatings, show the best ability to reduce friction losses of the sealing rings above 2000 rpm, which translates into a significant reduction in the engine resistance torque in the most frequently used crankshaft rotational speed range from 3200 to 3600 rpm. With regard to TiN coatings, assuming a 45% share of the friction losses per piston rings of the engine, it can be said that DLC1 coatings contribute to the reduction in friction losses of the rings by approximately 14.471%.
(2) TiAlN coatings perform best in the range of crankshaft rotational speeds below 2000 rpm for all engine oil temperatures considered. In this range, the difference in the values of the moments of resistance to motion of the engine for the TiN and TiAl coatings ranges from 2.507 to 3.334%.
(3) The TiN coating produces the greatest resistance to motion in relation to the TiAlN, CrN and DLC1 coatings, and the difference in these values for the temperature of 100 °C and the rotational speed of the crankshaft in the range from 3200 to 3600 rpm is follows: for the TiN and TiAlN coatings from 2.417 to 4.540%, and for TiN and DLC1 coatings from 4.174 to 6.512%.
(4) The TiN, TiAlN, CrN and DLC1 coatings show the ability to increase the engine resistance torque in the range from 800 to 1000 rpm for an oil temperature of 100 °C in relation to higher rotational speeds of the engine crankshaft.
(5) Coatings with a low roughness value contribute to the effective reduction in friction losses, especially of the TiAlN and DLC1 coatings. It is also highly dependent on the correlation between the hardness and surface texture after the running-in period. The process itself and the accuracy of the surface preparation for the coating are also important.
(6) Based on dry tribometric tests and with the use of oil mist, it was found that the lowest values of the friction coefficients have DLC1, TiAlN, CrN and TiN coatings. The value of the coefficient of friction closely depends on the conditions of the surface preparation for the applied coating, but also on the material used for the counter-sample.

All analyzed coatings produce similar waveforms of the engine resistance torque for the entire range of the considered engine rotational speeds. Their differentiation is subject to the value of the torque of resistance to movement of the engine for the entire range of engine rotational speeds. Assuming that the macro-shape of the sliding surfaces of the piston rings is very similar after the running-in period, it can be concluded that the changes in the torque of the resistance to movement of the engine are influenced by additional properties of anti-wear coatings, e.g., the ability to wet the surface with oil or the texture of the surface layers of the coatings. Certainly, it is an interesting topic for further research in order to determine the coefficients of correlation of oil cohesion and adhesion to the substrate for selected anti-wear coatings used in internal combustion engines.

## Figures and Tables

**Table 1:** Technical Data of the Aircraft Internal Combustion Piston engine Intended for the Bench Tests.

Engine Model	40i
Total engine capacity (cm^3^)	627
Cylinder diameter (mm)	75.5
Piston stroke (mm)	70.0
Compression ratio	8.5
Axial height of the upper sealing ring (mm)	1.50
Axial height of the lower sealing ring (mm)	1.75
Axial height of the scraper ring shelf (mm)	0.40
Distance between the upper ring and the piston crown (mm)	4.00
Distance of the upper ring from the lower ring (mm)	3.40
Distance of the lower ring from the scraper ring (mm)	5.20
Medium pressure of the upper sealing ring (MPa)	0.20
Medium pressure of the lower sealing ring (MPa)	0.20
Mean pressure of the scraper ring (MPa)	1.00

## Data Availability

Not applicable.
